# Liraglutide upregulates the *Cftr* gene and regulates the mucus transcriptome profile in Brunner's glands in mice

**DOI:** 10.1002/ctm2.70510

**Published:** 2025-10-27

**Authors:** Louise Marie Voetmann, Bidda Rolin, Rikke Kaae Kirk, Lotte Bjerre Knudsen, Myrte Merkestein, Jonas Ahnfelt‐Rønne, Anne Louise Kodal, Carsten Jessen, Asli Ozen, Charles Pyke, Axel Kornerup Hansen

**Affiliations:** ^1^ Department of Veterinary and Animal Sciences Faculty of Health and Medical Sciences University of Copenhagen Frederiksberg Denmark; ^2^ Research & Early Development Department Novo Nordisk A/S Måløv Denmark

**Keywords:** Brunner's glands, Cftr, GLP‐1, gut, incretin, intestinal barrier, mucus

## Abstract

**Background:**

The metabolic syndrome encompasses a state of inflammation and metabolic dysfunction, possibly mediated via a disturbed intestinal barrier. Glucagon‐like peptide‐1 receptor agonists (GLP‐1RAs), such as liraglutide, have shown promising anti‐inflammatory effects beyond glucose lowering and weight loss, but the underlying mechanism remains to be elucidated. We hypothesised that GLP‐1RAs improve the intestinal barrier function and overall inflammatory status by direct gene activation in mucus‐secreting Brunner's glands in the mouse duodenum, known for their high density of glucagon‐like peptide‐1 receptors (GLP‐1Rs).

**Methods:**

Using bulk RNA sequencing, in situ hybridisation, and immunohistochemistry, we analysed the change in the genetic phenotype of mouse Brunner's gland cells following GLP‐1R activation by liraglutide.

**Results:**

We show that liraglutide induces a novel and robust upregulation of the gene for the Cystic fibrosis transmembrane conductance regulator, *Cftr*, in Brunner's glands as a part of an overall genetic phenotype involved in ion channel activity, mucus secretion, and hydration via GLP‐1R activation. Additionally, we found a robust upregulation of the genes *Muc5b*, *Il33*, *Ren1*, and *Vldlr* in Brunner's glands.

**Conclusion:**

Collectively, our results imply an enhanced mucus response from Brunner's glands following GLP‐1R activation, which might play a role in the effect of GLP‐1.

## Graphical abstract

RNA sequencing, in situ hybridisation, and immunohistochemistry show that the glucagon‐like peptide‐1 receptor agonist liraglutide upregulates *Cftr, Muc5b*, *Il33*, *Ren1*, and *Vldlr* in Brunner's glands of mice.

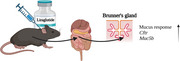

## 1 BACKGROUND

The gut is critical to overall health,^1^ and the intestinal barrier is a sophisticated system of integrating layers of cells with specialised functions needed to secure an adequate barrier function in the different intestinal compartments. A dysfunctional intestinal barrier causing chronic low‐grade inflammation is central to many diseases. This includes the metabolic syndrome affecting over a billion people globally ^2^ and that encompasses obesity, hyperlipidemia, insulin resistance, and hypertension, often combined with type 2 diabetes and cardiovascular diseases.^2^ Surgery bypassing the duodenum or exogenous administration of glucagon‐like peptide‐1 receptor agonists (GLP‐1RA) are potent ways to alleviate obesity, insulin resistance, and hyperglycaemia.^3^, ^4^ Furthermore, GLP‐1RAs (SELECT trial) have, similarly to bariatric surgery (SOS trial), shown to reduce the risk of major cardiovascular events.^5^, ^6^

GLP‐1RA with prolonged half‐life mimics the human insulinotropic gut hormone, glucagon‐like‐peptide‐1 (GLP‐1), and activates the glucagon‐like peptide‐1 receptor (GLP‐1R), distributed in many tissues in the body.^4^, ^7^ Native GLP‐1 is rapidly degraded by dipeptidyl peptidase 4 in the circulation, and its short half‐life supports a local role via activation of GLP‐1Rs in the gut.^7^ The most significant expression of GLP‐1R in the gut is found in the proximal part of the duodenum, in a network of mucus‐producing cells located in the submucosa, called Brunner's glands.^8^, ^9^ The secreted mucus is a part of the intestinal barrier protecting the epithelial cells against pathogens and the acidic stomach content entering duodenum. Brunner's gland cells are unique, as they are solely represented in the proximal part of the duodenum in mammals.^10^ Knowledge of Brunner's gland cells and function is primarily obtained through observational histology studies.^10^ Hence, there is a need to increase our understanding of the function of these cells. Although knowledge of Brunner's glands’ secretory response mechanism is limited, there is knowledge from other mucus‐secreting cells, such as goblet cells in the gut or lungs, which have been described more thoroughly.^11^, ^12^, ^13^, ^14^ We hypothesised that essential genes in the Brunner's glands related to the mucus response are upregulated by treatment with GLP‐1RA.

*Cftr* encodes the cystic fibrosis transmembrane conductance regulator (CFTR), that functions as a channel to secure the transport of chloride and bicarbonate ions, controlling the movement of water in the tissue, which is essential for proper mucus secretion and function.^12^, ^15^, ^16^, ^17^ In murine colon and human Caco‐2 cells, GLP‐1 has been shown to directly affect the expression of *Cftr* via adenosine 3′,5′‐cyclic monophosphate (cAMP) signalling.^18^ In Brunner's glands, GLP‐1 also upregulates cAMP.^19^ Mucus is primarily water bound to mucins, with additional factors such as electrolytes, lipids, and bactericidal factors.^1^, ^10^, ^11^, ^16^ *Muc5b* encodes a gel‐forming mucin, mucin 5b, which is best described in the respiratory tract. Here, *Muc5b* is essential for eliminating pathogens and maintaining immune homeostasis.^13^, ^14^ In the intestine, it is present in Brunner's glands and at low levels in the colon.^20^ *Cftr* and *Mucb5* are, therefore, essential mucus secretory factors,^13^, ^14^, ^21^ and the Brunner's glands may be highly relevant for their expression and subsequent mucus secretion. It has been shown that administration of the GLP‐1 receptor agonist liraglutide to a mouse colitis model induced transcriptional upregulation of the genes *Ccl20, Il33* and *Muc5b* in Brunner's glands, which alleviated colitis.^22^

We hypothesised that GLP‐1RA activates a mucus response in Brunner's glands of mice, and we, therefore, used a stepwise approach applying RNA sequencing to characterise the effect of GLP‐1R activation in Brunner's glands, and by the subsequent use of immunohistochemistry, in situ hybridisation analysis, and GLP‐1R‐deficient mice to show that the primary readouts, *Cftr* and *Muc5b*, were upregulated in the Brunner's glands upon stimulation with the GLP‐1RA liraglutide.

## 2 METHODS

### 2.1 Materials availability

Liraglutide (270 nm/mL) and vehicle (pH = 7.4; 50 mmol/L phosphate; 70 mmol/L sodium chloride;  .05% polysorbate 80) and GLP‐1 coupled anti‐sense‐oligonucleotide (ASO) targeting *Malat1* (289 µM ASO GLP‐1 conjugated peptide; 8.05 mM sodium phosphate dibasic; 1.96 mM potassium dihydrogen phosphate; 140 mM sodium chloride) were produced at Novo Nordisk, Måløv. The GLP‐1R knockout (KO) mouse was custom‐bred at Taconic, Denmark. The studies did not generate new, unique reagents.

### 2.2 Study approval

All studies were approved by the Council Animal of Experimentation under the Danish Ministry of Food, Fisheries, and Agriculture, and conducted in agreement with the Danish Animal Experimentation Act (Legislation Order No 63 of January 19 2024) and the European Union Directive (2010/63/EU) (licenses #2013‐15‐2934‐00784 and #2017‐15‐0201‐01215), and it is reported in accordance with the ARRIVE guidelines.^23^

### 2.3 Animal studies

Mice used for the gene regulation studies were C57BL6/JRj (Janvier, France) and C57BL6/JBomTac (Taconic, Denmark) (B6J) male mice, aged 8–10 weeks upon arrival. The nephrotoxic serum nephritis (NTN) mice were female Crl:CD1(ICR) (CD1) (Charles River, Germany) mice, and 7 weeks at arrival. In addition, C57BL/6J‐*Glp1r^tm1Ddr^* (Taconic) (GLP‐1R KO) were used for the study to confirm GLP‐1R internalisation and dependency. 24 male diet‐induced obese (DIO) C57BL/6JCrl mice and 8 C57BL/6JCrl low‐fat diet (LFD mice) (Charles River, France) were used for the DIO studies. For further information, see Supplementary Methods. Upon arrival to the Animal Unit, Novo Nordisk, Måløv, mice had an acclimatisation period of a minimum of 1 week. Animals were housed 5–10 per cage. For DIO and GLP‐1R KO mice, separated pair housing with a partitioning wall was used, in temperature (22 ± 2°C) and humidity (50 ± 20%) controlled rooms. The circadian rhythm was 12 h light:12 h dark (Lights on at 06:00 am), with ad libitum access to food and water, depending on the study (see study overview Figure S1). To identify the grouped housed mice, their ear were marked after the acclimatisation period. Bedding and enrichment materials (nest, gnawing stick, climbing robe) were exchanged every week, and caretakers inspected animals daily. In all studies, humane endpoints were applied in cases when the animals showed signs of permanent suffering, pain, or fear, while loss of appetite associated with the analogue was expected. However, only a maximum of 20% body weight loss was allowed. If weight loss was above 10%, mice received saline s.c. to avoid dehydration and were closely monitored.

### 2.4 Method details

More detailed methods are described in Supplementary Methods.

### 2.5 Formalin perfusion

Once mice were fully anaesthetised by inhaling isoflurane (Forene®, Abbott, Germany; induction 5%, maintenance 2% isoflurane,  .7 L/min N2O,  .3 L/min O2), a laparotomy was performed by an abdominal midline incision and exposure of the thoracic cavity by an incision along the sternum. Mice were perfused via pumps (Masterflex, Pennsylvania, United States) with  .9% NaCl with heparin (1 U/mL) through the heart at a rate of 5 mL/min in 2 min, followed by 4% formalin perfusion at the same rate in 5 min and tissue was collected and stored in 10% neutral buffered formalin for later histological analysis.

### 2.6 Gene expression analysis

Note that  .5 cm of the proximal duodenum was exercised and immediately collected in RNAlater or snap‐frozen and stored in –20°C or –80°C, respectively, until further analysis. The tissue was homogenised in RTL lysis buffer with beta‐mercaptoethanol (β‐ME) (Qiagen Inc, Netherlands), and purified using the Rneasy mini kit with DNase treatment (Qiagen Inc). The RNA quantity was assessed by Nanodrop and Qubit (ThermoFisher Scientific, Massachusetts, United States), while the quality was measured using the Agilent 2100 Bioanalyser with the RNA 6000 Nano kit (Agilent, Germany). The total amount of RNA isolated was approximately 30–50 ng/µL per duodenum sample. Gene expression was analysed using RNA bulk sequencing, qPCR, or in situ hybridisation, details described in Supplementary Methods.

### 2.7 Histology

Also,  .5 cm of the proximal duodenum was excised, stored in 10% neutral buffered formalin for 48 h, and processed for formalin embedding. Sections were stained using in situ hybridisation, immunohistochemistry, or a combination of both methods, see details in Supplementary Methods.

### 2.8 Quantification and statistical analysis

The sample sizes were calculated in power analysis based upon prior experience performing similar studies. The mice were randomised into dose groups and blinded when conducting qPCR and RNA‐sequencing, including histological semi‐quantification. Except for RNA sequencing, all data were analysed using GraphPad Prism version 9.0.1 (GraphPad Software, San Diego, USA), and *p*‐values ≤  .05 and *q*‐values ≤  .05 were considered significant. All data are shown as mean ± standard deviation unless otherwise stated. Data were tested for equal variance by the Brown‐Forsythe test, and Gaussian distribution by the D'Agostino–Pearson test. When applicable, two‐tailed parametric analysis was used. The detailed analysis appears in legends. Data that did not show equal variance or normal distribution were log‐transformed. In cases where log‐transformed data did not pass parametric test criteria, Mann–Whitney *U* or Kruskal–Wallis tests were applied. For multiple analysis, *p*‐values were corrected by False‐Discovery Rate (FDR). Specific analysis details and exact sample sizes (biological replicates) are depicted in figure legends.

## 3 RESULTS

### 3.1 *Cftr* and *Muc5b* were significantly upregulated in Brunner's glands in mice after acute liraglutide exposure

**FIGURE 1:**
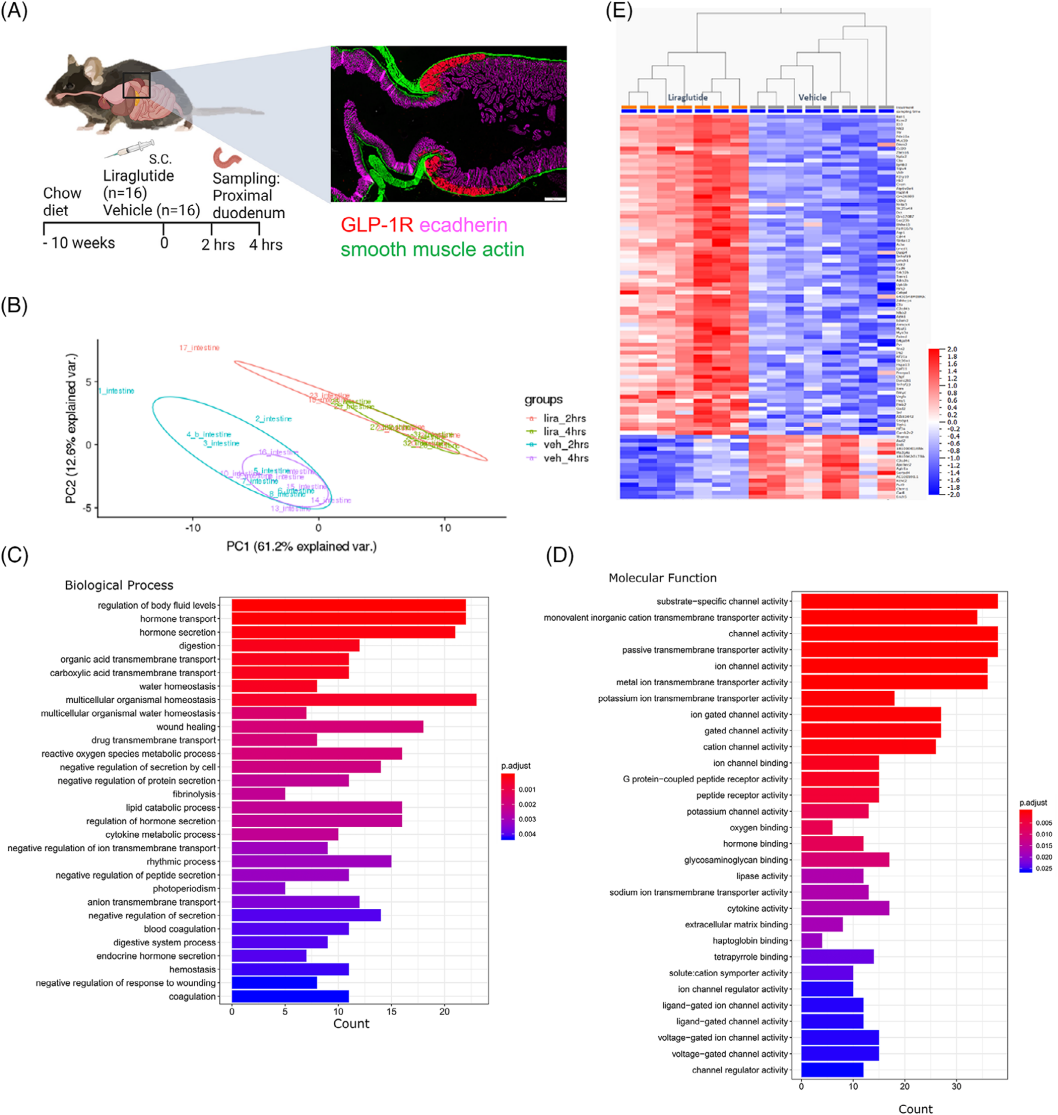
Brunner's gland cell regulation following glucagon‐like‐peptide‐1 (GLP‐1) receptor (GLP‐1R) activation. Schematic illustration of study set‐up. B6/J male mice were dosed with either vehicle (veh) or liraglutide (lira) (A). The proximal part of the duodenum containing the GLP‐1R expressing Brunner's cells was sampled for RNA sequencing following 2 and 4 h of exposure (*n* = 8 per group). Principal component (PC) analysis revealed that groups could be separated due to treatment at both sampling points (B). Gene set enrichment analysis of the 2 h time point (C) and the 4 h time point (D). Heatmap of significantly differentially regulated genes between the treatment groups following 4 h of liraglutide exposure (E).

To elucidate the cellular dynamics of Brunner's glands' following GLP‐1R activation, we dosed 16 chow‐fed male B6J mice with a long‐acting GLP‐1 analogue, liraglutide, and 16 similar mice with the vehicle. We sampled the proximal part of the duodenum containing Brunner's glands for RNA sequencing 2 and 4 h later, based on previous experience (Figure 1A).^20^ Using principal component analysis, there was a clear separation of liraglutide and vehicle‐treated groups, indicating an effect of liraglutide in the proximal duodenum (Figure 1B). Liraglutide regulated 682 genes after 2 h and 1212 genes after 4 h of exposure compared to the vehicle.

To understand the biological consequences of the genes regulated by liraglutide, we conducted a gene set enrichment analysis of genes regulated at both time points.^24^ Liraglutide enriched pathways involved in regulating body fluid levels, glycosaminoglycan binding, and secretory and ion channel pathways (Figure 1C and D). The gene regulation was most pronounced at the 4‐h time point in agreement with earlier studies; hence, further work was focused on this time point.^20^ At the 4‐h time point, the differential expression analysis after FDR correction showed 149 significantly regulated genes (Figure 1E).

**FIGURE 2:**
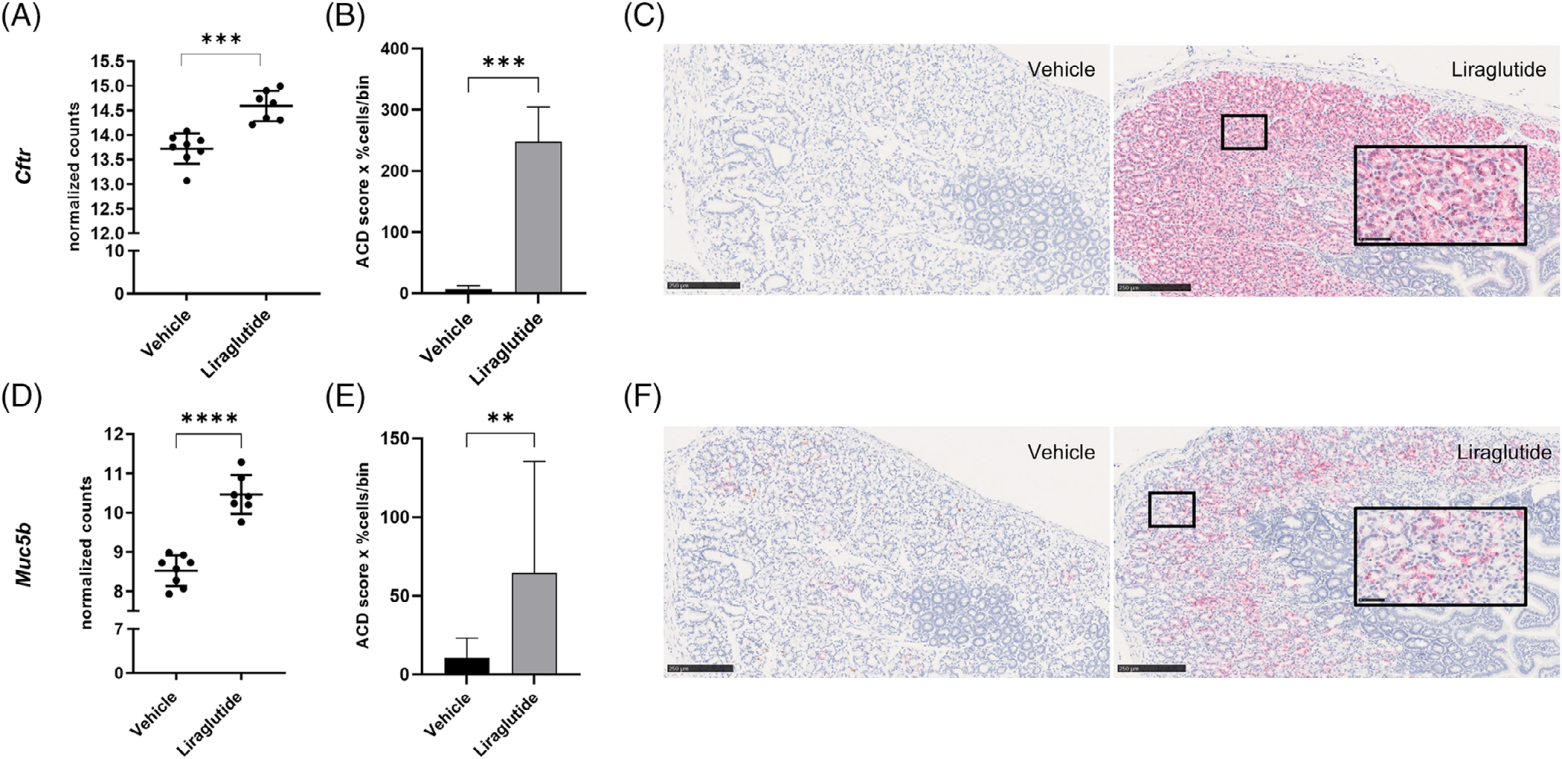
Liraglutide upregulates the expression of genes encoding cystic fibrosis transmembrane conductance regulator (Cftr) and mucin 5b (Muc5b). Liraglutide significantly upregulated *Cftr* (A) and *Muc5b* (D) expression after 4 h exposure in an RNA sequencing study; unpaired two‐tailed *t*‐test, FDR corrected using the Benjamini, Krieger and Yekutieli procedure. A follow‐up study was conducted in 4 h dose B6/J mice (*n* = 7 or 8 per group). ISH analysis of *Cftr* showed a significant Brunner's gland specific upregulation in the liraglutide treated mice (B, C), Mann–Whitney test. Furthermore, ISH of *Muc5b* confirmed a similar significant upregulation in Brunner's glands following liraglutide exposure (E, F), unpaired two‐tailed *t*‐test on log‐transformed data. Data are shown as mean ± SD. *p  <  .05, **p  <  .01, ***p  <  .001, ****p  <  .0001. The *Cftr* analysis was done on *n* = 6–8/group (1 section per mouse, ROI = Brunner's glands). ACD score (1–4) = [1 × (cell fraction with 1–3 dots/cell) + 2 × (cell fraction with 4–9 dots/cell) + 3 × (cell fraction with 10–15 dots/cell) + 4 × (cell fraction with > 15 dots/cell)].^93^ Representative images for each group of ISH for Mm‐*Cftr* and Mm‐*Muc5b*; scale bar 100 µm. ISH, in situ hybridisation, ROI = region of interest.

The gene expression analysis revealed an upregulation of the mucus‐related genes *Cftr* (2‐fold, Figure 2A) and *Muc5b* (5‐fold, Figure 2D), following 4 h of liraglutide exposure. Therefore, we selected the upregulated *Cftr* and *Muc5b* transcripts as primary readouts for the subsequent studies. Additionally, the genes *Ren1*, *Vldlr*, and *Il33*, which are not yet well described in mucus cells, were found to be significantly upregulated following liraglutide and were, therefore, included as secondary readouts in the in situ hybridisation analysis to investigate if they were related to Brunner's gland cells or a subset of other cells in the duodenum.

### 3.2 In situ hybridisation confirmed increased *Cftr* and *Muc5b* expression in Brunner's glands

The vulnerable epithelial cells of the duodenum rely on a proper mucus barrier to prevent a potential acidic insult following gastric emptying. Here, mucus is secreted primarily from Brunner's glands and a limited number of goblet cells.^10^, ^20^ Both hormones and neuronal stimulation have been shown to generate a mucus secretory response from Brunner's glands.^25^, ^26^ Due to its high GLP‐1R density, which is well conserved between mouse and man, the idea that GLP‐1 plays a significant role in Brunner's gland is highly likely.^9^, ^27^, ^28^

Therefore, we investigated if the observed upregulations of the primary readout genes were restricted to Brunner's gland cells. We collected the upper part of the duodenum 4 h after liraglutide administration for histological analysis. The regulation of *Cftr* and *Muc5b* was evaluated by in situ hybridisations, which enabled us to evaluate the precise location of the transcripts, and to quantify the amount in the proximal duodenal tissue sampled (Figure 2B–F). In all Brunner's glands cells, a distinct upregulation of the *Cftr* gene was observed in the liraglutide‐treated mice (Figure 2C). Using quantitative image analysis, we found the *Cftr* gene to be significantly upregulated in Brunner's glands in liraglutide‐dosed mice (Figure 2B). Similarly, the upregulation of *Muc5b* was restricted to Brunner's glands cells, and a significant upregulation of the transcript was found in the liraglutide‐treated group (Figure 2E and F). It should be noted that the *Cftr* transcript was expressed by more Brunner's gland cells as compared to *Muc5b* in liraglutide‐treated mice.

To better understand the role of *Cftr* and *Muc5b* in Brunner's glands, we included immunohistochemistry for the translated proteins, i.e., the cystic fibrosis transmembrane conductance regulator (CFTR) channel and mucin 5b, to visualise their distribution in the tissue (extended Figure 1; Supplementary Material). The CFTR channel was present in the apical membrane in all the Brunner's gland cells, while mucin 5b was present in the cytoplasm of a subset of cells in variable amounts. These results agree with existing knowledge, that is, in a mucus‐secreting cell, the mucins are stored in vesicles, the so‐called mucin granules, in the cytoplasm, which are transported to the apical membrane, where mucins are secreted by exocytosis and expanded via CFTR channel‐mediated hydration.^10^, ^17^

### 3.3 Liraglutide upregulations of *Ren1*, *Vldlr* and *Il33* are specific for Brunner's gland

**FIGURE 3:**
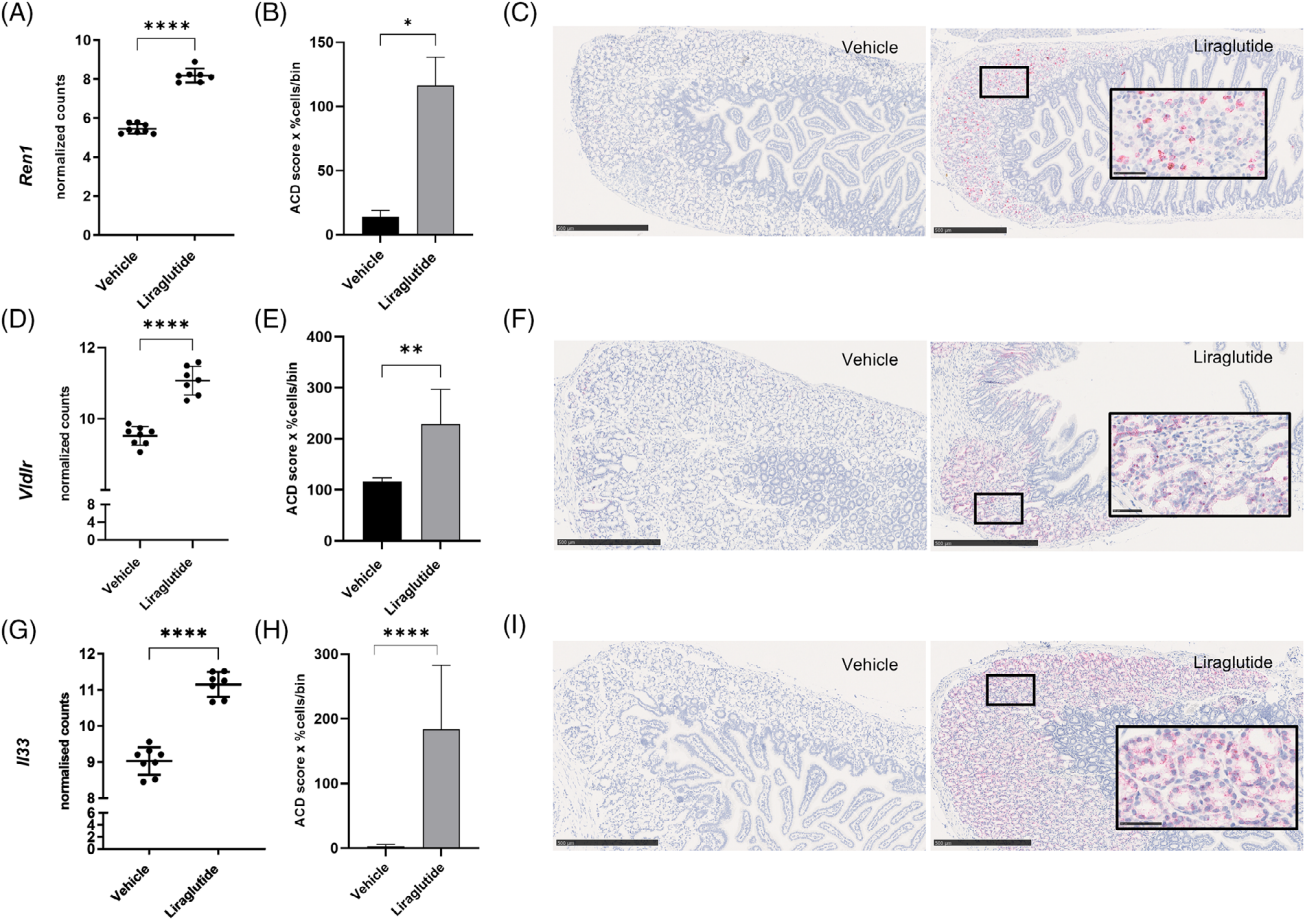
Liraglutide upregulates the expression of genes in Brunner's glands encoding renin (Ren1), very low‐density lipoprotein receptor (Vldlr) and interleukin 33 (Il33). RNA sequencing data showed a significant upregulation of *Ren1*, *Vldlr* and *Il33* following acute exposure to liraglutide (A, D, G). Unpaired two‐tailed *t*‐test, False‐Discovery Rate (FDR) corrected using the Benjamini, Krieger and Yekutieli procedure. ISH confirmed that all three transcripts were significantly upregulated following 4 h of liraglutide exposure (*n* = 7 or 8 per group) in Brunner's glands. Il33 (H) was tested using unpaired two‐tailed *t*‐test, Vldlr (E) and Ren1 (B) tested using Mann–Whitney test. Data are shown as mean ± SD, *p  <  .05, **p <  .01, ***p  <  .001, ****p  <  .0001. The *Ren1* analysis was done on *n* = 4/group. Representative images of the ISH of Mm‐Ren1 (C), Mm‐Vldlr (F) and Mm‐Il33 (I) for the groups; scale bar 100 µm (1 section per mouse, ROI = Brunner's glands). ACD score (1–4) = [1 × (cell fraction with 1–3 dots/cell) + 2 × (cell fraction with 4–9 dots/cell) + 3 × (cell fraction with 10–15 dots/cell) + 4 × (cell fraction with > 15 dots/cell)].^93^ ISH, in situ hybridisation; ROI, region of interest.

The *Ren1* transcript was by far the most upregulated gene (21‐fold, Figure 3A–C) in our initial dataset. *Ren1* encodes renin, a part of the homeostatic renin‐angiotensin system involved in various physiological processes, for example, cell proliferation, inflammation, water and electrolyte absorption and secretion, motility and blood flow.^29^ Additionally, renin acts independently of the renin‐angiotensin system via the (pro)renin receptor encoded by *Atp6ap2*. Interestingly, the (pro)renin receptor has been found in mucin vesicles, which might control cellular and intracellular pH.^30^, ^31^ The *Vldlr* gene, which was 3.5‐fold upregulated (Figure 3D–F), encodes the very low‐density lipoprotein receptor, which plays a vital role in triglyceride metabolism, and affects the crypt‐villus homeostasis in the gut via the reelin signalling pathway.^32^, ^33^ The *Il33* gene, which was 4.5‐fold upregulated (Figure 3G–I), encodes the interleukin 33 cytokine, which has been reported to be involved in anti‐inflammatory T helper cell type 2 responses as a part of the innate immune system and as an intestinal alarmin.^34^, ^35^ *Il33* has also been implicated in goblet cell differentiation and found to be co‐localised with mucins in mucus cells.^36^, ^37^ In situ hybridisation of the three transcripts confirmed a Brunner's gland cell‐specific significant upregulation in liraglutide‐treated mice (Figure 3A–I). *Ren1* seemed to be expressed in a limited subset of the Brunner's gland cells, while *Vldlr* and *Il33* were expressed in a larger number of the Brunner's gland cells. We only observed the *Ren1* signal in Brunner's gland epithelial cells and therefore concluded that the Ren1 gene is likely implicated in the secretory function of this gland.

### 3.4 *Muc5b* was expressed in more Brunner's gland cells than *Ren1*

As the expression of *Muc5b* and especially *Ren1* was identified in a subset of Brunner's gland cells, we investigated the presence of different subtypes of Brunner's gland cells and whether any cells showed co‐expression of these two transcripts. To explore a possible co‐expression of the transcripts in Brunner's gland cells, we incorporated duplex in situ hybridisation that detects two transcripts simultaneously at single‐cell resolution. Our analysis confirmed that *Ren1* was expressed in a limited number of Brunner's gland cells. However, only a few cells co‐expressed the two genes. *Muc5b* was found to be expressed in more Brunner's gland cells compared to *Ren1* (Figure S2).

### 3.5 GLP‐1 activation internalises the GLP‐1R in Brunner's glands

**FIGURE 4:**
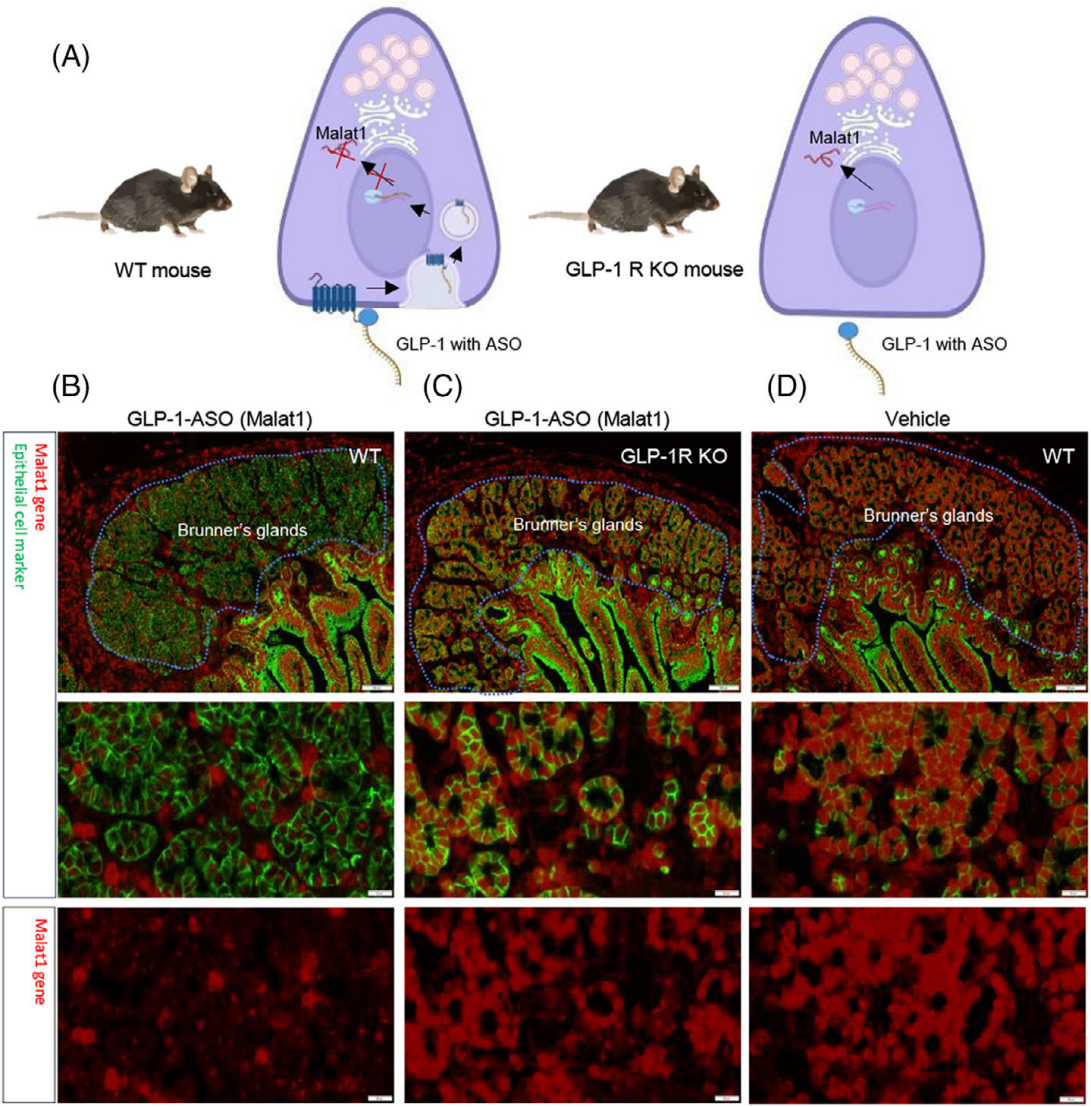
Glucagon‐like‐peptide‐1 (GLP‐1) conjugated antisense oligonucleotide silencing Malat1 expression delivered by GLP‐1 receptor (GLP‐1R) internalisation. Schematic illustration shows the antisense oligonucleotide (ASO) delivery principle via GLP‐1R activation and internalisation following GLP‐1 binding in wild type (WT) mice and GLP‐1R knockout (KO) mice (A). Seventy‐two hours after GLP1‐ASO administration, the *Malat1* was silenced in WT mice (B) but not in GLP‐1R KO mice (C) or vehicle dosed WT mice (D) (*n* = 6 per group). Representative images of fluorescence in situ hybridisation of *Malat1* (red) and immunohistochemistry staining of epithelial cells (green) for the groups; scale bar 100 µm.

Besides Brunner's glands, the GLP‐1R is expressed in enteroendocrine cells, Paneth cells, intraepithelial lymphocytes, and neurons in the submucous plexus and the myenteric plexus in the intestine.^8^ To provide evidence of direct activation of the GLP‐1R in Brunner's glands following GLP‐1 stimulation, we conducted a study using in vivo gene silencing as previously described.^38^ Here, a GLP‐1 conjugated antisense oligonucleotide targeting *Malat1* was delivered via GLP‐1R internalisation into Brunner's glands in mice (Figure 4A). After 72 h, there was a significant silencing of *Malat1* in wild‐type (WT) mice following GLP‐1R internalisation and *Malat1* silencing (Figure 4B). In GLP‐1R KO mice, there was no GLP‐1 receptor present. Hence, the antisense was not delivered internally in the Brunner's glands, and no *Malat1* silencing was observed (Figure 4C). Correspondingly, there was no *Malat1* silencing in vehicle‐treated mice (Figure 4D). In the same experiment, *Malat1* silencing in the pancreas was also examined as a positive control. The GLP‐1 conjugated antisense oligonucleotide targeting *Malat1* reduced *Malat1* expressing selectively in the beta cells of the pancreas, which are known to express GLP‐1R (data in repository). Based on the experiment, we can conclude that the GLP‐1R in the Brunner's glands is activated and internalised following GLP‐1 administration. We only observed *Malat1* silencing in Brunner's glands and not anywhere else in duodenum.

### 3.6 The upregulations of *Cftr*, *Muc5b*, *Ren1* and *Il33* following acute exposure to liraglutide are GLP‐1R dependent

**FIGURE 5:**
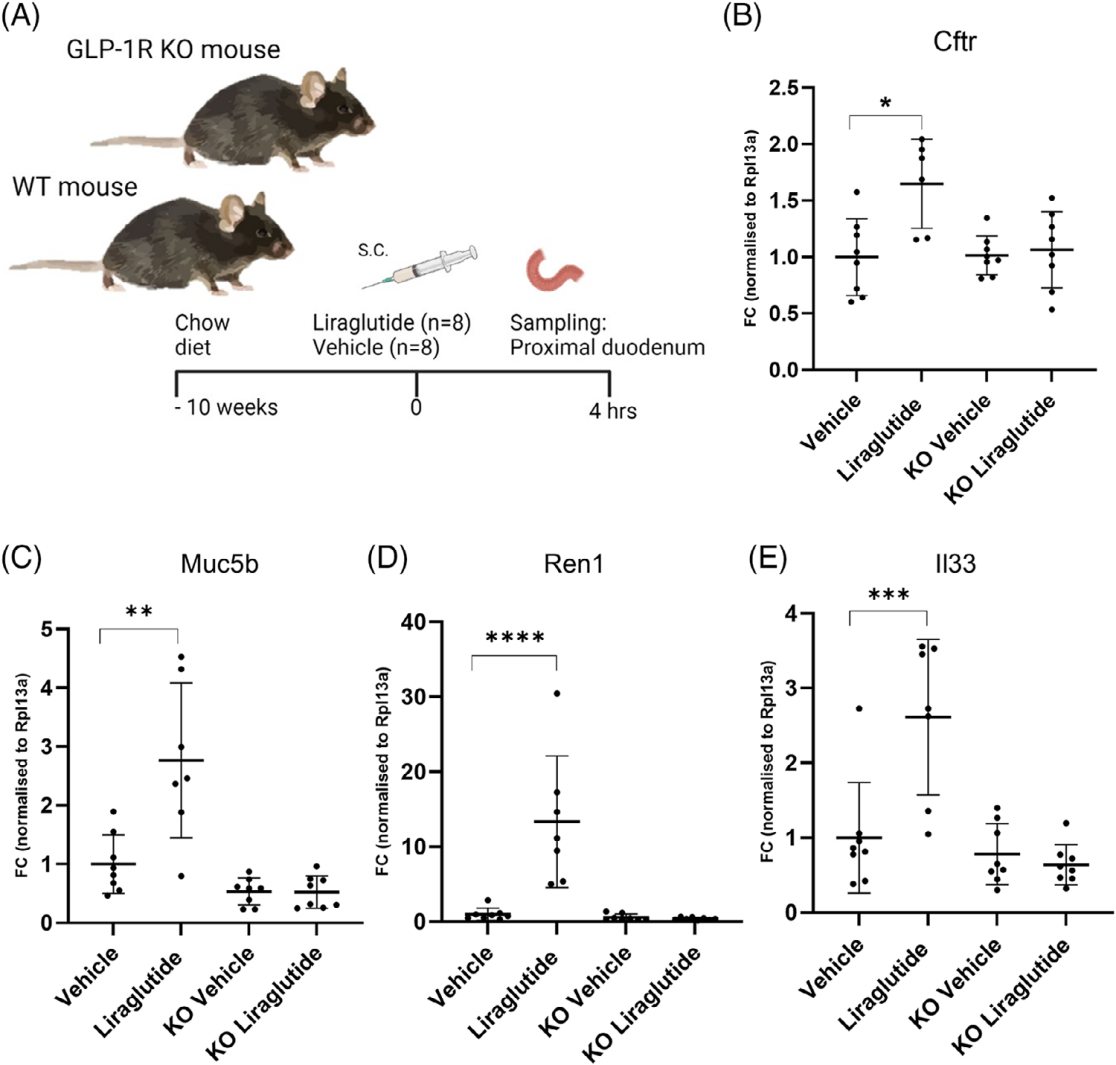
Gene expression following liraglutide administration depends on activation of the GLP‐1 receptor (GLP‐1R). The study set‐up included WT B6/J mice and GLP‐1R KO on the same genetic background, which was exposed to liraglutide or vehicle for 4 h (*n* = 8). Proximal duodenum was sampled for qPCR analysis (A). *Cftr* (B), *Muc5b* (C), *Ren1*(D) and *Il33* (E) were significantly upregulated in the WT group treated with liraglutide. No gene regulation was observed in the GLP‐1R KO mice. Data are shown as mean ± SD. One‐way ANOVA Sidak corrected post hoc test (B), Kruskal–Wallis with Dunn's post hoc test (C), two‐way ANOVA Sidak corrected on log‐transformed data (D, E). *p  <  .05, **p  <  .01, ***p  <  .001, ****p <  .0001. One animal was excluded from the liraglutide group due to insufficient housekeeping gene levels.

Next, we included transgenic GLP‐1R KO mice in a similar acute study to determine if the observed effect depended on a functional GLP‐1R (Figure 5A). We included a subset of the genes of interest in the qPCR analysis and found a significant upregulation of all investigated genes in our WT mice following 4 h of liraglutide exposure. The effect was ablated in the GLP‐1R KO mice (Figure 5B–E). Hence, we concluded that the effect observed in Brunner's glands following liraglutide was receptor dependent. Additionally, we sampled proximal duodenal tissue from GLP‐1R‐deficient mice to investigate the expression of *Muc5b* and *Cftr* in Brunner's glands by immunohistochemistry. The encoded proteins were expressed similarly to the WT mice (Figure S3).

### 3.7 Liraglutide's acute effects on Brunner's glands persist in a mouse prediabetes and obesity model

In both mice and humans, a diet high in fat and carbohydrates has been associated with adverse changes in the mucus layer throughout the intestine.^39^, ^40^ In the small intestine, CFTR‐mediated mucus, and liquid secretion is important for limiting bacterial exposure to secure the effective uptake of nutrients without risking an insult to the intestinal barrier. Long‐term consumption of a high‐fat diet reduces the expression of *Cftr*, causing dysfunctional mucus and an influx of pathogens into the intestinal mucosa and circulation.^12^, ^17^, ^41^ To investigate if the potential positive effect of liraglutide on Brunner's glands mucus function is maintained in a patient‐relevant model, we included mice fed a high‐fat diet for 28 weeks. Here, the expressions of our primary and secondary gene readouts (*Cftr*, *Muc5b*, *Ren1*, *Vldlr*, *Il33*) by qPCR analysis after 4 h of liraglutide exposure were evaluated. We included the gene *Atp6ap2*, also present in our sequencing dataset, encoding the (pro)renin receptor‐interacting at the cell membrane with renin as we hypothesised that renin is involved in vesicular acidification in Brunner's gland mucus cells via *Atp6ap2*.^30^, ^31^

**FIGURE 6:**
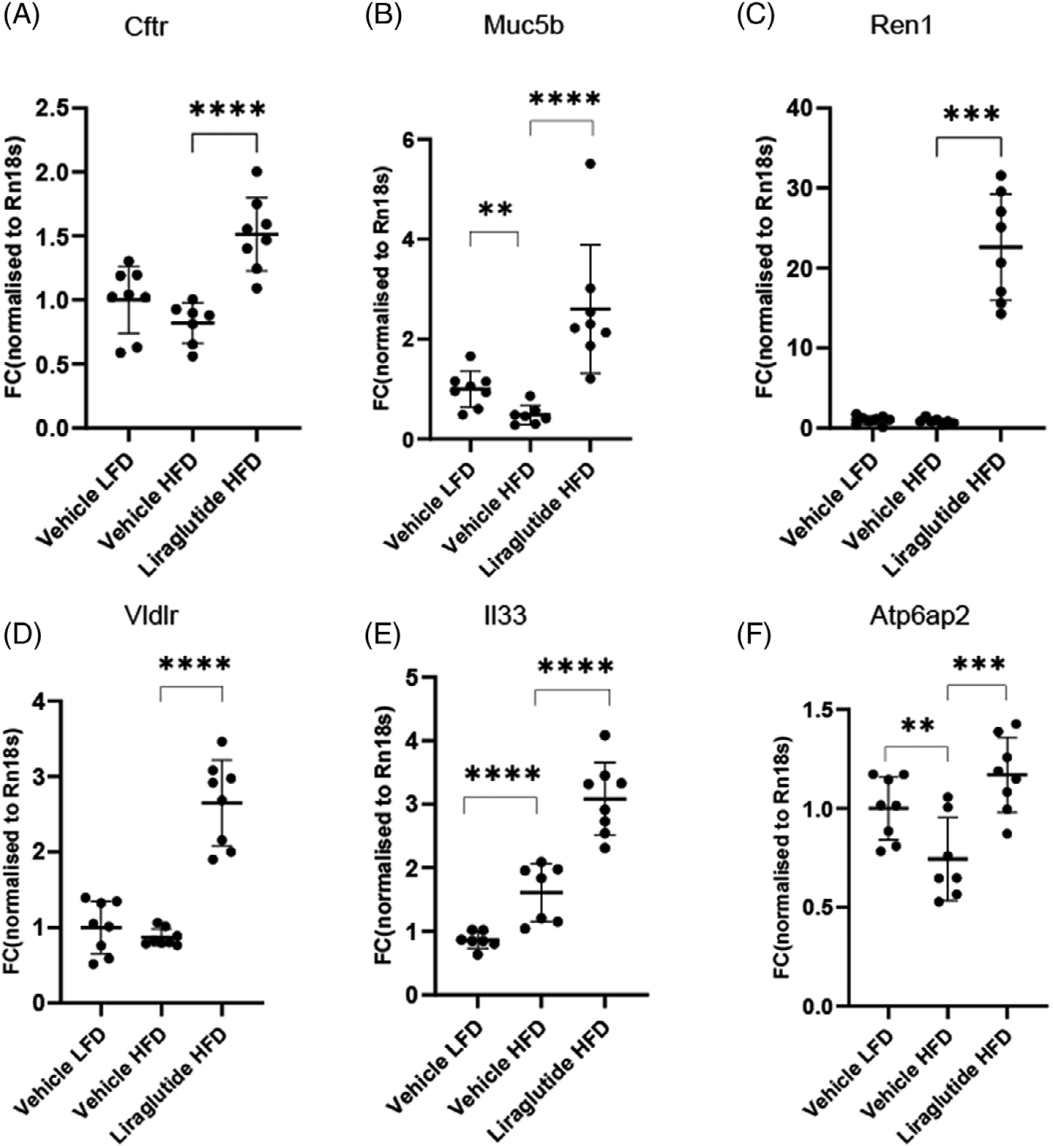
Upregulation of genes of interest maintained in diet‐induced obese (DIO) mice. The study set‐up included low‐fat‐fed (LFD) and high‐fat‐fed (HFD) DIO mice on the same B6/J genetic background, exposed to liraglutide or vehicle for 4 h (*n* = 8/group). The proximal duodenum was sampled for qPCR analysis. *Cftr* (A), *Muc5b* (B), *Ren1* (C), *Vldlr* (D), Il33 (E), and *Atp6ap2* (F) were significantly upregulated in DIO mice treated with liraglutide compared to vehicle‐treated DIO mice. Furthermore, a significant change in genes expression comparing DIO and LFD mice for *Muc5b* (B), *Il33* (E) and *Atp6ap2* (F) was found. Data are shown as mean ± SD. Two‐way ANOVA FDR corrected (A, D, F). Two‐way ANOVA FDR corrected on log‐transformed data (B, E), Kruskal–Wallis with Dunn's post hoc test (C). *p  <  .05, **p  <  .01, ***p  <  .001, ****p  <  .0001. LFD, low‐fat diet; HFD, high‐fat diet; DIO, diet‐induced obese; Lira, liraglutide; Veh, vehicle.

All genes included in the analysis were upregulated following 4 h of liraglutide exposure in the high‐fat‐fed mice (Figure 6A–F). When comparing gene expression levels in vehicle dosed mice, no significant effect following a high‐fat diet for 28 weeks was found for the *Cftr*, *Ren1* and *Vldlr* transcripts (Figure 6A, C, and D). However, *Muc5b* and *Atp6ap2* transcripts were significantly reduced (Figure 6B and F). Notably, the high‐fat diet increased the expression of *Il33*, which increased further following liraglutide exposure (Figure 6E). Liraglutide was previously found to upregulate *Il33* acutely, while long‐term exposure decreased the expression over time.^20^ Collectively, we can conclude that the Brunner's glands effects following acute exposure to liraglutide are maintained in our model of prediabetes and obesity. Furthermore, high‐fat diet negatively affects the mucus‐related genes *Muc5b* and *Atp6ap2*.

### 3.8 *Cftr* and mucin 5b protein levels were increased in Brunner's glands following long‐term exposure to liraglutide in a chronic kidney disease model

**FIGURE 7:**
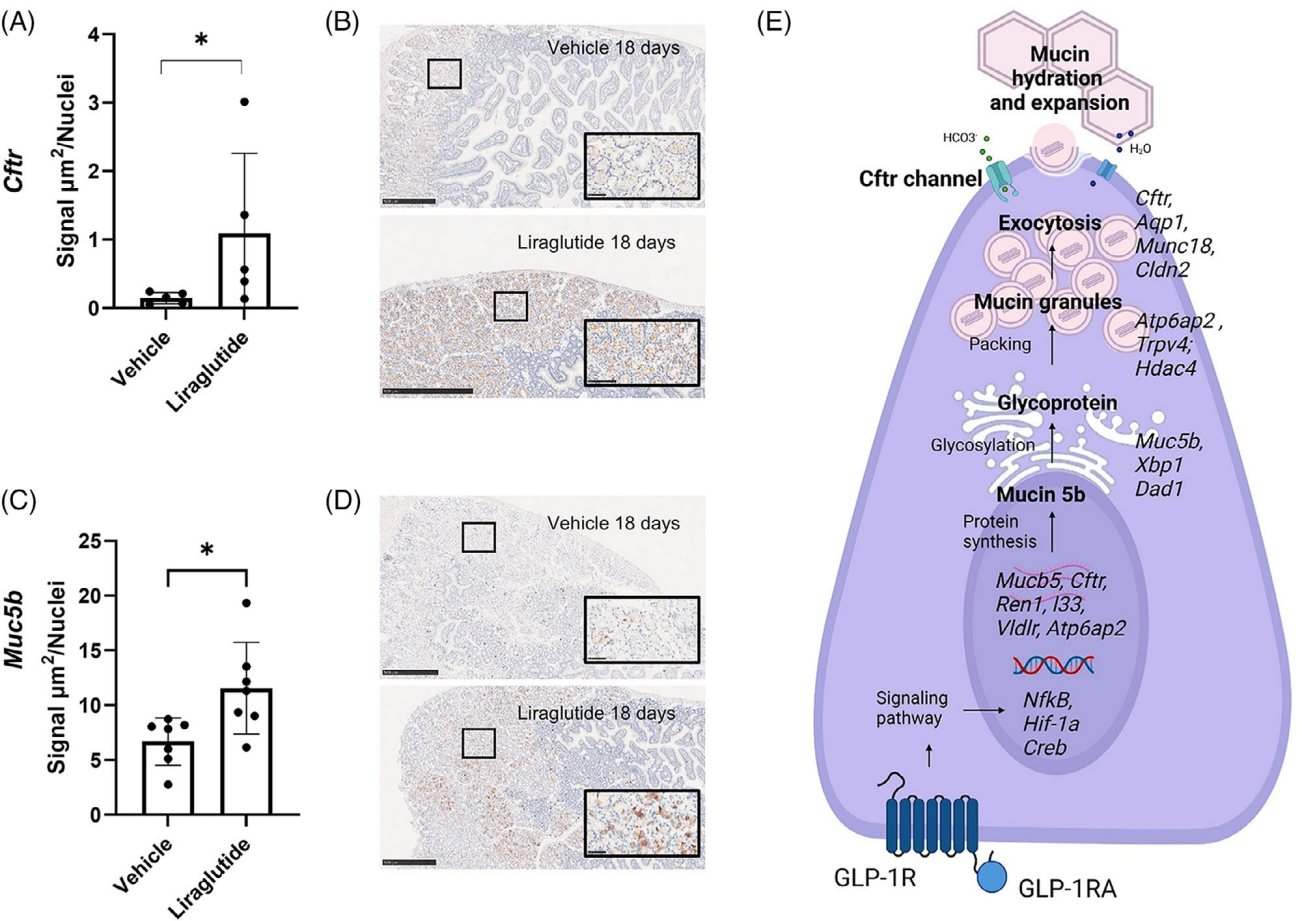
Liraglutide upregulates protein expression of the Cftr channel and mucin 5b following 18 days long‐term exposure. CD‐1 female mice were dosed daily with liraglutide or vehicle for 18 days and duodenum was sampled for histology. All groups received NTS intravenously on days 4 and 5. Using a virtual imaging system (VIS) custom‐made application, the output of the area of immunohistochemistry signal per cell was detected and quantified (*n* = 5–7 per group, 1 section per mouse, ROI = Brunner's glands). The signal area per cell was significantly upregulated in the liraglutide treated group for the Cftr channel (A) and the mucin 5b (C). Representative images for the included groups of the quantified protein amount stained by DAB, Cftr (B) and mucin 5b (D); scale bar 500 µm. Data are represented as mean ± SD, unpaired two‐tailed *t*‐test on log‐transformed data (A) and unpaired two‐tailed *t*‐test (C). ROI, region of interest; NTS, nephrotoxic serum.

Treatment with liraglutide improves hallmarks of chronic kidney disease and it reverses the regulation of several fibrosis and inflammation associated genes as in human patients.^42^ Therefore, we investigated the effect of long‐term exposure to liraglutide on mucin 5b and CFTR protein level in an NTN mouse model of chronic kidney disease. Here, female CD‐1 mice were treated with liraglutide for four days before the induction of glomerulonephritis by sheep anti‐rat nephrotoxic serum (NTS) administration and the following 14 days.^42^ Immunohistochemistry with subsequent imaging analysis was used to visualise and quantify the levels of the two proteins encoded by the *Muc5b* and *Cftr* genes. The level of the CFTR channel was significantly increased in the apical membrane of Brunner's gland cells in the liraglutide treated group (Figure 7A and B). Similarly, a significantly higher amount of mucin 5b was present in Brunner's glands of liraglutide treated mice (Figure 7C and D). As such, the mucus related effect of liraglutide in Brunner's glands seems to be more than an acute phenomenon, as the effect is maintained following sub‐chronic exposure to the drug.

In summary, these results highlight the role of GLP‐1 as an important regulator of the mucus response in Brunner's gland. Liraglutide increases the expression of several genes involved in different mucus production and secretion stages, as depicted in Figure 7E. In addition, we observed a GLP‐1‐mediated upregulation of *Il33*, *Ren1*, *Vldlr*, genes that are not well described in relation to a mucus response. Finally, we have provided evidence for at least two subsets of Brunner's gland epithelial cells’ existence.

## 4 DISCUSSION

The aim of this study was to characterise the effect of GLP‐1R activation in Brunner's glands. As hypothesised, we demonstrate a strong effect of GLP‐1RA on mucus synthesis and secretion genes, including a robust upregulation of the genes *Cftr* and *Muc5b* in Brunner's gland cells of the mouse duodenum following acute exposure. This effect was maintained following long‐term treatment. The upregulated expressions of *Cftr* and *Muc5b*, as well as *Ren1*, *Vldlr*, and *Il33* transcripts, were found to be GLP‐1R dependent, as no effect was observed in Brunner's glands following liraglutide treatment of GLP‐1R KO mice. Subsequently, we demonstrated that the GLP‐1R is activated in Brunner's glands following GLP‐1 administration.

Our results demonstrate a direct effect of GLP‐1RA on Brunner's glands mucin production and secretion, and it is likely that this effect will improve the barrier function of the intestinal mucus layer. This supports the hypothesis that the anti‐inflammatory effects of GLP‐1RAs could be at least partly explained by an improved function of the gut epithelial barrier.^8^, ^20^ As observed in metabolic syndrome patients, excessive nutrient intake stresses the gut and impairs immune responses and gut homeostasis, leading to chronic gut inflammation. This may drive systemic inflammation by translocation of gut‐derived pathogenic factors affecting various metabolic organs.^43^, ^44^ Targeting inflammation has proven effective clinically, as shown in the large CANTOS trial in patients with metabolic syndrome, including type 2 diabetes and cardiovascular disease. Here IL‐1B antagonism via IL‐1B targeting monoclonal antibody Canakinumab improved Beta‐cell function, glycaemia, and reduced high‐sensitivity C‐reactive protein (hsCRP) by 39% and cardiovascular events by 15%, although an increased risk of infection warrants caution for further exploration of this treatment modality.^45^, ^46^ The GLP‐1RA class of effective anti‐diabetic and weight loss drugs has recently been shown to reduce the risk of cardiovascular events by 20% and similarly reduce the hsCRP by 39% (SELECT trial) ^47^ and 43.5% (STEP trial) ^48^ with an acceptable safety profile.^49^, ^50^ Furthermore, the hsCRP levels have been shown to remain improved compared to placebo 1 year after treatment withdrawal (STEP trial), although the positive effect on weight was diminished.^51^ This highlights an extended anti‐inflammatory effect in addition to the beneficial effects of weight loss per se.

One theory that has been brought forward proposing the anti‐inflammatory effects of GLP‐1RAs to be a potential improvement of the dysfunctional intestinal barrier associated with the metabolic syndrome via GLP‐1R‐mediated effects on gut microbiota composition, mucosal healing, motility, and local inflammation.^20^, ^52^, ^53^, ^54^, ^55^ In our studies, both transcriptomic and histological analyses strongly supported a direct effect of GLP‐1RA treatment on *Cftr* and *Muc5b* expression in Brunner's gland. Both are genes involved in mucus secretion. The role of *Muc5b* in mucus secretion and function has been extensively described in the lung, where *Muc5b* has a critical role in ensuring the effective clearing of pathogens.^13^, ^14^ Interestingly, genetic variants of *Muc5b* have been associated with type 2 diabetes.^56^ We found a reduced *Muc5b* expression in the DIO mouse, which could be reversed by GLP‐1RA treatment. The CFTR channel is vital for proper fluid and electrolyte balance in a range of organs, including the lung and intestine.^12^, ^15^, ^16^, ^17^ A dysfunctional CFTR channel, as seen in patients with cystic fibrosis, causes an altered intestinal milieu due to decreased bicarbonate and water secretion. The ensuing impaired mucus function negatively affecting bacterial clearance and neutralisation of gastric acid, causing malabsorption, and decreased antimicrobial activity, bacterial overgrowth, hyper‐inflammation, and increased intestinal permeability.^57^, ^58^, ^59^, ^60^, ^61^, ^62^, ^63^ While the *Cftr* gene is regulated by a complex network of upstream elements, ^64^ it is less complex for *Muc5b*, which is transcriptionally regulated by thyroid transcription factor‐1 (TTF‐1) and GATA‐6 transcription factors.^65^

Intriguingly, decreased expression of *Cftr*, leading to decreased bicarbonate secretion and increased susceptibility of the intestinal mucosa to barrier dysfunction, has been reported following obesity and diabetes.^41^, ^66^, ^67^, ^68^ Additionally, in human intestinal cells, high levels of IL‐1B downregulate *CFTR* expression, which has been linked to an increased pro‐inflammatory response.^69^, ^70^ Our data point to direct regulation of the *Cftr* gene by GLP‐1RA and indicate that this may be vital for ensuring optimal properties of the mucus layer, including the hydration and neutralisation of the duodenal epithelium upon entrance of acidic stomach content.^71^ The upregulation of *Cftr* could also improve local inflammation indirectly alongside effects mediated by intraepithelial lymphocytes.^55^

In the respiratory system, GLP‐1RA has been shown to regulate *Muc5b* expression in airway epithelial cells, and this effect has been hypothesised to benefit patients with chronic obstructive pulmonary disease and cystic fibrosis.^72^ This potential mode‐of‐action is supported by a substantial number of studies that have reported improved mucus clearance function, reduced bacterial burden and improved overall lung function following GLP‐1RA treatment.^72^, ^73^, ^74^ However, none of the studies reported a direct effect of GLP‐1R activation on the CFTR channel, although our studies show that this could be an underlying effect and indicates a similar mode of action as seen in the lungs, namely that GLP‐1RA improves the intestinal barrier function.

*Il33* encodes the interleukin 1 superfamily interleukin 33 (IL‐33) released by cells as a danger‐associated molecular pattern (DAMP) to initiate the generation of CD8^+^ memory T cells and to signal stress, damage, or cell death to make T helper cells, mast cells, eosinophils and basophils produce type 2 cytokines.^75^, ^76^ The latter causes severe pathological changes in mucosal organs.^77^ Intracellularly, IL‐33 is a nuclear factor ^78^ known to interact with a histone methyltransferase ^79^ and NF‐κB.^80^ IL‐33 is also known to alleviate Alzheimer‐like symptoms in APP/PS1 mice.^81^ *Vldlr* encodes a cell surface receptor (VLDL‐R) that binds very low‐density lipoproteins (VLDL), which mediates lipid uptake in especially heart, skeletal muscle, and adipose tissue cells, but unless during stress *Vldlr* only plays a minor role in lipid metabolism mice.^82^ Knockout or overexpression of *Vldlr* in *Ldlr* (low‐density lipid receptor) knockout mice leads to higher or lower serum triglyceride levels, respectively, compared to the single knockout mice.^81^ *Vldlr* knockout mice show cerebellar hypoplasia and disrupted neuronal layering, as VLDL‐R is essential for guiding neurons to their correct positions in the developing brain.^83^ VLDL‐R is also implicated in atherosclerosis,^84^, ^85^ and a receptor for certain viruses.^86^ *Ren1* encodes renin, which initiates the renin‐angiotensin‐aldosterone cascade from the kidneys to regulate blood pressure, fluid balance, and electrolyte homeostasis.^87^ It is also known to be expressed in other tissues like the submandibular gland in certain strains of mice.^87^ *Ren1 k*nockout mice suffer from kidney vascular abnormalities, and hyper‐ or hypotension, depending on the genetic context.^88^

The limitations of our study could be the lack of a specific Brunner's glands in GLP‐1R KO mouse. Therefore, we cannot exclude that additional GLP1R activation in other cells and tissues can affect Brunner's glands following GLP‐1 dosing. Furthermore, it should be noted that the potential effect of GLP‐1RA in the respiratory system could be a mouse phenomenon as the GLP‐1R is present in various cells in rodents but so far only in airway vascular smooth muscle cells in humans.^9^, ^27^, ^28^, ^74^ Notably, Vara et al. demonstrated surfactant secretion from human type II pneumocytes in vitro upon GLP‐1R stimulation.^89^ Furthermore, it would have been helpful to elucidate the gut permeability. However, we have previously published that the FITC‐dextran method is unreliable in DIO mice,^90^ and, therefore, we decided not to include it in this study. Finally, it is a limitation that we did not elucidate the functional effects of a changed regulation of *Il33*, *Vldlr* and *Ren1*.

While the study of upstream elements for the *Cftr* gene is complicated, it would be relevant in the future to study the effect of the upstream elements of *Muc5b* by liraglutide treatment of mice deficient of TFF‐1^65^ or GATA‐6 ^91^ Future studies should also include a further functional elucidation of the effect of the GLP‐1RA impact on the Brunner's glands. In live non‐obese mice tests for gut permeability, lipid uptake and blood pressure could be made. More complex studies on cystic fibrosis could be made in knock‐in mice with a human *Cftr* mutation, such as the *ΔF508*.^92^ Duodenal histology could include PAS staining, immunofluorescence co‐staining, and single‐cell RNA sequencing.

## 5 CONCLUSION

We have demonstrated an until now underappreciated role of GLP‐1RA‐mediated activation of Brunner's glands leading to mucus secretion, which is partly facilitated via upregulation of the central genes *Muc5b* and *Cftr* and potentially assisted by the genes with less well‐defined roles in intestinal barrier function (*Il33*, *Vldlr* and *Ren1)*. This effect was shown following acute dosing of liraglutide and thus independent of the metabolic improvements following chronic treatment. Moreover, the effect was maintained in obesity and a chronic kidney disease model, including following long‐term dosing. A GLP‐1RA improvement of the gut mucosa would potentially reduce local and systemic inflammation associated with metabolic dysfunction in patients and could represent an important piece to the puzzle explaining the positive pleiotropic effect on inflammation of GLP‐1Ras.

## AUTHOR CONTRIBUTIONS

All authors listed were involved in concept development and data analysis/interpretation. L.M. Voetmann designed and performed in vivo studies, gene expression analysis, histological characterisation, and imaging analysis. The ASO‐GLP‐1 study was managed by M. Merkestein, and the subsequent histological analysis by J.A. Rønne. C. Jessen and A.L.B. Kodal did the ASO‐GLP‐1 peptide manufacturing. A.I. Ozen performed all bioinformatic analyses. L.M. Voetmann and A.K. Hansen wrote the manuscript. All authors have revised and approved the final manuscript.

## CONFLICT OF INTEREST STATEMENT

L.M. Voetmann, B. Rolin, R.K. Kirk, L.B. Knudsen, C. Jessen, A.L.B. Kodal, J.A. Rønne, A.H Ozen, and C. Pyke are employed by Novo Nordisk A/S. L.B. Knudsen, B. Rolin, and C. Pyke are minor shareholders as part of employment at Novo Nordisk A/S. A.K. Hansen declares that he has collaborated with the pharmaceutical industry and received funding from this source, as described on https://ivh.ku.dk/english/employees/?pure%20=%20en/persons/107126.

## ETHICS STATEMENT

All animal experiments were performed in compliance with internationally recognised guidelines for the use of laboratory animals (licenses #2013‐15‐2934‐00784 and #2017‐15‐0201‐01215) and received approval from the Danish Animal Experimentation Committee under the Danish Ministry of Food, Fisheries and Agriculture. All studies were conducted by trained and licensed personnel at Novo Nordisk A/S.

## Supporting information



Supporting Information

## Data Availability

Source data is available at https://osf.io/whs4j/?view_only%20=%2029202ba4160f46f68edeb2b47f4c0ea7 and upon request to corresponding author.
