# Supplementary material for: Liraglutide upregulates the Cftr gene and regulates the mucus transcriptome profile in Brunner's glands in mice
Source: Clin Transl Med. 2025 Oct 27;15(11):e70510. doi: 10.1002/ctm2.70510 (PMC12559683; doi:10.1002/ctm2.70510)
Supplement: Supplementary file 1 — Supporting Information [file CTM2-15-e70510-s001.docx]

**Supplemental material**

## *Supplementary methods*

### Code availability

This paper does not report original code.

Data analysis are provided in the methods. Any further questions or reasonable requests should be directed to the lead of contact.

### Studies

#### Initial characterization study exploiting RNA sequencing

32 C57BL6/JBomTac mice (Taconic, Denmark) fed a chow diet (Altromin 1324, Brogaarden, Denmark) were weight stratified in two groups, receiving a single subcutaneously (SC) injection of liraglutide (1mg/kg, n=8/grp) or vehicle (pH = 7.4; 50 mmol/L phosphate; 70 mmol/L sodium chloride; 0.05% polysorbate 80, n=8/grp) all from Novo Nordisk, Måløv, Denmark. At study termination, the mice were 12 weeks old. Two and four hours after dosing, mice were anaesthetized by inhaling isoflurane (induction 5%, maintenance 2 % isoflurane, 0.7 L/min N2O, 0.3 L/min O2), and euthanized by cervical dislocation. The upper 0.5 cm of the duodenum was immediately collected, snap-frozen and stored at -80 °C until further analysis. Before mice were euthanized, retro-orbital blood was sampled into EDTA coated tubes and plasma separated (4 °C; 5 min; 6000xG) for exposure analysis using AlphaScreen technology (Perkin Elmer Denmark).

#### Study to confirm Brunner’s gland cells specific gene regulation following acute liraglutide exposure

20 C57BL6/JRj mice (Janvier, France) fed a chow diet (Altromin 1324, Brogaarden, Denmark), aged 11 weeks at the day of study, were used. The study design paralleled the above but only with the 4 hours’ time point. At study termination, mice were anaesthetized (induction 5%, maintenance 2 % isoflurane, 0.7 L/min N2O, 0.3 L/min O2), and formalin perfusion was performed, see method details. Immediately after perfusion, the first 0.5 cm of the duodenum was collected and stored in 10% neutral buffered formalin for histological analysis.

#### Study of GLP-1R Internalization

12 C57BL6/JBomTac and 6 C57BL6/J GLP-1R KO (C57BL/6-*Glp1r^tm1Ddr^*) (Taconic, Denmark) male mice fed a chow diet (Altromin 1324, Brogaarden, Denmark) aged 12 weeks at study initiation ^1^. Six C57BL6/J and six GLP-1R KO were dosed SC with 1000 nmol/kg of ASO-GLP-1 dissolved in phosphate-buffered saline (PBS) and six C57BL6/J with vehicle (PBS). Seventy-two hours after dosing, the mice were anaesthetized (induction 5%, maintenance 2 % isoflurane, 0.7 L/min N2O, 0.3 L/min O2), and formalin perfusion was performed, and proximal duodenum and pancreas were sampled for histological analysis.

#### Study to confirm GLP-1R dependency

16 C57BL6/Jrj mice (Janvier, France) and 16 C57BL6/Jax GLP-1R KO (C57BL/6-Glp1r<tm1Ddr>) mice (Taconic, Denmark) were used ^1^. The mice were 16 weeks at study termination. The two groups of mice were weight stratified to the treatment groups (liraglutide or vehicle, n=8/grp) according to the study design described earlier. Four hours after injection, mice were anaesthetized by inhalation of isoflurane (induction 5%, maintenance 2 % isoflurane, 0.7 L/min N2O, 0.3 L/min O2), euthanized by cervical dislocation, and the first 0,5 cm of the duodenum was immediately collected and stored in RNAlater (Thermofisher) at -20°C for qPCR analysis.

#### Study to address retained effects in DIO mice

24 DIO C57BL/6JCrl mice and 8 C57BL/6JCrl LFD mice (Charles River, France), 28 weeks of age at termination, were used. On the day of the study, LFD mice and half of the DIO mice had an SC injection of vehicle or liraglutide accordingly to the previous studies (n=8/grp). Four hours after dosing mice were anesthetized by inhalation of isoflurane (induction 5%, maintenance 2 % isoflurane, 0.7 L/min N2O, 0.3 L/min O2) and euthanized by cervical dislocation. The first 0.5 cm of the duodenum was collected in RNAlater and stored at -20°C for qPCR analysis.

#### 18 days liraglutide exposure in NTN mice

24 Crl:CD-1 female mice (Charles River, France) on a chow diet (Altromin 1324, Brogaarden, Denmark), 11 weeks of age at study termination, were used. Liraglutide or vehicle was given prophylactically in an 18-day period (n=8/grp). The dose of liraglutide was up titrated from 0.3 mg/kg to 1 mg/kg in the first three days. On study days 4 and 5, mice received one dose of 100µL nephrotoxic serum (NTS) from sheep intravenously in the tail vein (Batch 530-5T-E, Probetex, San Antonio, USA). The mice were weighed every day and dosing was based on actual body weight at the time of dosing. On the last day of the study, mice were anesthetized by inhalation of isoflurane (induction 5%, maintenance 2 % isoflurane, 0.7 L/min N2O, 0.3 L/min O2). Blood from the retro-orbital vein was collected before mice were perfusion fixed, and the upper part of the duodenum was collected in 10% neutral buffered formalin for histological analysis. The blood samples were collected in EDTA coated tubes and plasma separated (4 °C; 5 min; 6000xG) for exposure analysis using AlphaScreen technology (Perkin Elmer, Denmark).


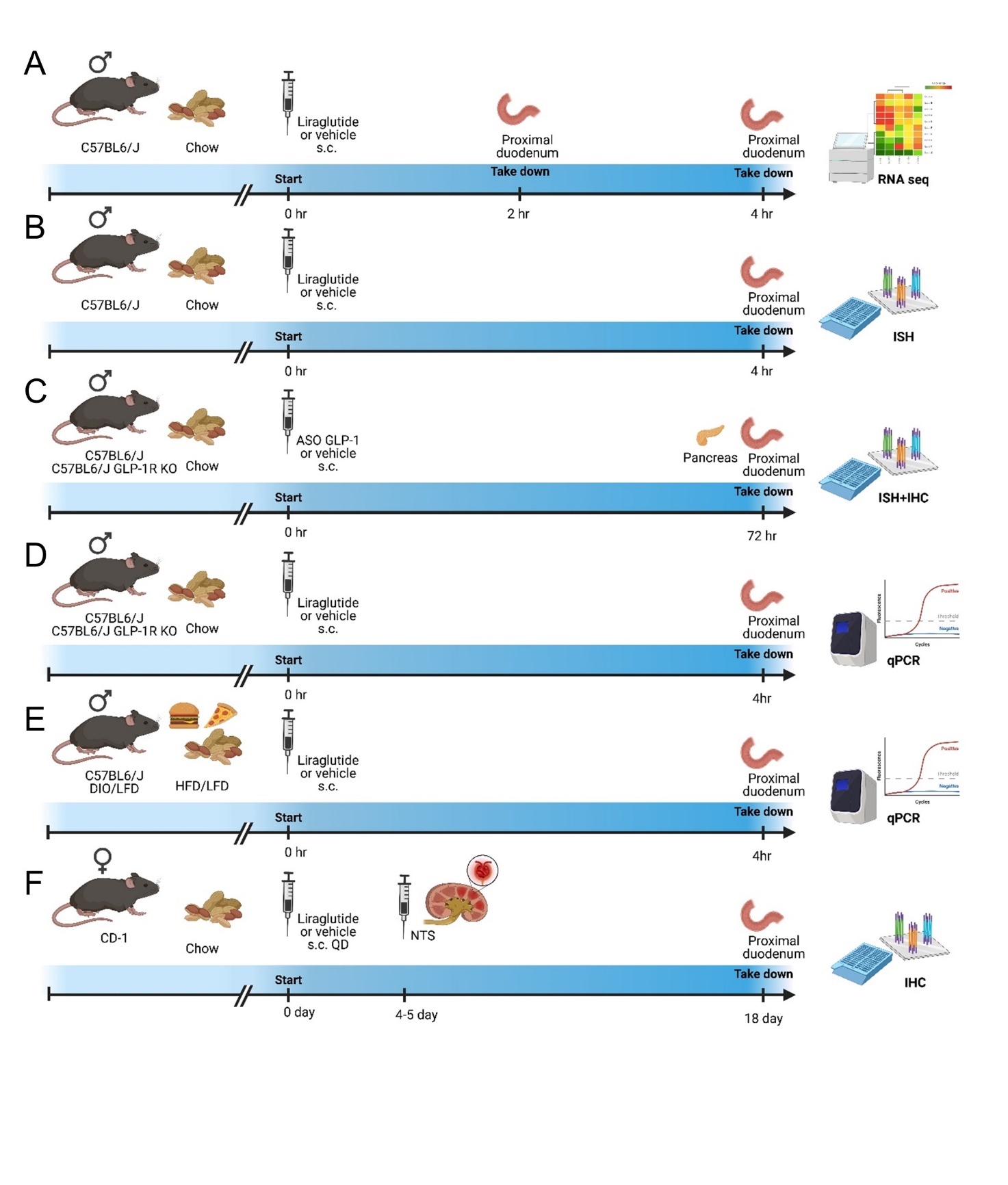


### S.Fig. 1: Overview of mouse studies

*A)* *Initial characterization study exploiting RNA sequencing of the proximal duodenum in 32 C57BL6/JBomTac mice (Taconic, Denmark) fed a chow diet (Altromin 1324, Brogaarden, Denmark) dosed with vehicle or liraglutide (270 nmol/kg). B) 4 hours study to confirm Brunner’s gland cells specific gene regulation following acute liraglutide exposure in 20 C57BL6/JRj mice (Janvier, France) fed a chow diet (Altromin 1324, Brogaarden, Denmark). C)* *Study to confirm GLP-1R internalization in Brunner’s glans in 12 C57BL6/JBomTac and 6 C57BL6/J GLP-1R KO (C57BL/6-Glp1r^tm1Ddr^) (Taconic, Denmark) male mice fed a chow diet (Altromin 1324, Brogaarden, Denmark) dosed with either ASO-GLP-1 dissolved in PBS (1000 nmol/kg) or with vehicle. D)* *Study to confirm GLP-1R dependency in 16 C57BL6/Jrj mice (Janvier, France) and 16 C57BL6/Jax GLP-1R KO mice (Taconic, Denmark) dosed with vehicle or liraglutide (270 nmol/kg). E)* *Study to address retained effects in 24 DIO C57BL/6J mice and 8 C57BL/6J LFD mice (Charles River, France). F)* *18 days liraglutide exposure study in NTN mice. 24 CD-1 female mice (Charles River, France) on a chow diet (Altromin 1324, Brogaarden, Denmark), 11 weeks of age at study termination, were used. Liraglutide or vehicle was given prophylactically in an 18-day period (n=8/grp) once daily (QD). The dose of liraglutide was up titrated from 0.3 mg/kg to 1 mg/kg in the first three days. On study days 4 and 5, mice received one dose of 100µL nephrotoxic serum (NTS)ensuring the development of glomerulonephritis. RNA seq : RNA sequencing, ISH: In situ hybridization, IHC: Immunochemistry.*

### Method details

#### Peptide synthesis

The peptide was supplied by Apigenex s.r.o. ^2^. It was synthesized using standard Fmoc based solid phase synthesis on a Rink Amide AM resin (Novabiochem) as a C-terminal amide and purified by RP-HPLC at Apigenex s.r.o.. Standard Fmoc amino acids were used in addition to Fmoc-2-aminoisobutyric acid-OH (Fmoc-Aib-OH).

Peptide sequence: H**Aib**EGTFTSDVSSYLEEQAAKEFIAWLVKGGPSSGAPPPSC-NH_2_

#### Conjugation of Malat1 antisense oligonucleotide with GLP-1RA

The ASO against Malat1 was obtained from Axolabs GmbH at a purity of >85%. The sequence is based on that reported by Ämmälä and colleagues ^2^ using locked nucleic acids (LNA) instead of constrained ethyl (cET).

ASO sequence: 5’aminohexyl- TCA**G*^m^C*A***T*T*C*T*A*A*T*A*G* **^m^C *A*G**-3’

*represents phosphonothioate linkages

Bold: represents LNA

^m^C = 5-methylcytosine

The reaction was performed with 6*275 nmol ASO.

The ASO (275 nmol, 1,1 mM, 250 µl) was mixed with SPDP (succinimidyl 3-(2-pyridyldithio)propionate) in DMF (50 mM, 90 µl), phosphate buffer (1 M, 60 µl, pH 8) and reacted for around 2 h with shaking.

The six reactions (6*400 µl) were combined and diluted to 24 ml with HEPES (100 mM, pH 7.5). It was concentrated in 3K Amicon filters and washed three times with HEPES buffer to remove excess reagents. The retentate was collected and quantified (1600 nmol, 270 µl) from the absorbance at 260 nm.

All the material (1600 nmol, 270 µl) was mixed with peptide in DMF (3100 nmol, 310 µl) and TEA buffer (200 mM, 300 µl, pH 8.5), EDTA (200 mM, 60 µl), and water (260 µl) and reacted overnight at room temperature. The conjugate was purified by anion exchange (Agilent HPLC) (DNApac 4*250 mm column; solvent A: 100 mM NH_4_OAc in 30% MeCN pH 8; solvent B: 100 mM NH_4_OAc in 30% MeCN pH 8, 1.5 M NaBr; 0-100%, 2.5 ml/min.) followed by RP-HPLC (Agilent HPLC) (Clarity Oligo-RP 3 µm 50*4.6 mm; solvent A: 100 mM TEAA; solvent B: 100 mM TEAA in MeCN; 10-90%, 3 ml/min.). The pure fractions were combined, and the compound was freeze dried. Lastly the freeze-dried conjugate was reconstituted in freshly prepared PBS(1x) and buffer exchanged in a 3K Amicon filter with PBS(1x) to remove any leftover TEAA. The correct mass was confirmed by LC-MS (Waters Xevo G2-XS QTof connected to a Waters Acquity UPLC H Class) and the yield was measured by UV absorbance at 260 nm.

Yield (484 nmol, 605 µM, 29%). LCMS: Calc. 10639.73; found [M - 4H]^4-^ 2658.52 , [M - 5H]^5-^ 2126.59.

#### qPCR

For qPCR analysis, the cDNA was prepared using Script reverse transcription supermix (Bio-Rad Laboratories Inc, California, USA), and the qPCR was run with Taqman fast advanced master mix (Applied Biosystems, California, USA). Rn18s (Mm03928990_g1) or Rpl13a (Mm05910660_g1) were used as housekeeping genes and the following TaqMan primers; GLP-1r (Mm00445292_m1), Muc5b (Mm00466391_m1), Cftr (Mm00466391_m1), Ren1 (Mm02342887_mH), Il33 (Mm00505403_m1), Vldlr (Mm00443298_m1), Atp6ap2 (Mm00510396_m1), and muc2 (Mm01276696_m1). All qPCR analyses were performed in duplicates.

#### RNA-sequencing

The RNA concentration of all samples was measured using a Qubit with a HS RNA kit, and ~1000ng of total RNA was rRNA depleted, DNase treated and purified using New England Biolabs Inc.’s rRNA depletion kit (NEB-6350) and Agencourt RNAClean XP Beads(A63987, Beckman Coulter Life Sciences) with a fragmentation time of 7 minutes. Following depletion, the RNA concentration was remeasured using Qubit (ThermoFisher Scientific, Massachusetts, United States) RNA High Sensitivity kit, and 5 µl of each sample was processed for library preparation. Libraries were prepared using New England Biolabs Inc.’s Next Ultra II RNA Library Prep Kit for Illumina (NEB-E7775L) and Next Multiplex Oligos for Illumina (NEB-E6440S) following the manufacture’s protocol (NEB #E7770S/L, #E7775S/L 24/96 reactions Version 1.0, chapter 2). Denaturation and library dilution was performed as recommended in the Illumina protocol “Denature and Dilute Libraries Guide” (#15048776 v02). Libraries were sequenced with the High-Output Kit v2.5 (75 cycles, 20024906, Illumina) using a Nextseq 550 (Illumina, California, United States). All samples with a RIN ≥6.4, measured by Agilent Bioanalyzer with the High Sensitivity DNA kit (Agilent, California, United States), was accepted for sequencing. Instrument data in BCL format was demultiplexed and converted to FASTQC files using bcl2fastq v2.20.0.422 as instructed by the vendor. Based on the quality check performed by FastQC (version 0.11.7) ^3^, reads were quality trimmed using FASTX-Toolkit version 0.0.13 ^4^, where reads were trimmed for the 5’end 9nt, quality trimmed the 3’ end to Q30, and quality filtered to minimum base quality 100. Filtered reads were then aligned to GRCm38 v80 Ensembl Mus musculus genome using STAR RNA-Seq aligner version 2.6.0a ^5^. The resulting count matrix and the sample annotation sheet were further analyzed in R ^6^. The count matrix was filtered for gene counts higher than 5. Count matrix was annotated using the Ensembl Biomart gene IDs and names ^7^, where duplicated genes were cleaned by keeping the higher expressed gene pair. After the QC step, one sample with very low normalized counts and outliers in the PCA were removed from the analysis. Differential gene expression analysis was performed with DEseq2 ^8^ on the raw counts where pairwise comparisons for all conditions were obtained. Variance stabilization transformation was applied to the raw counts for normalization (<https://www.rdocumentation.org/packages/DESeq2/versions/1.12.3/topics/vst>). The fold-change was done using these normalized counts. Differentially expressed genes with a Benjamini and Hochberg adjusted P≤0.05 (5% False Discovery Rate) were regarded as statistically significantly regulated, where a negative log fold change value was considered a down-regulation, and a positive log-fold change an up-regulation. Significantly regulated genes were then used in the gene set enrichment analysis was performed with clusterProfiler ^9^ with “GO.db” ^10^ as the molecular functionality database, and msigdb mus musculus database for gene IDs. Benjamini-Hochberg adjusted p-value cutoff (FDR<0.05) and a value cutoff (0.1) were applied for significance, and the top 30 significantly enriched pathways were visualized using enrichGO function.

#### Histology

The proximal 5 mm of the duodenum was excised, and tissue was fixated for 48 hours in 10 % neutral buffered formalin, processed on a tissue processor (Leica ASP300s, Leica Biosystems, Germany) and embedded in paraffin. Paraffin blocks were sectioned into 4,5 µm slides and placed on Superfrost Plus glass slides (Thermo Fisher Scientific, USA).

#### Immunohistochemistry

The immunohistochemistry was fully automated and performed on the Ventana Ultra Discovery (Roche Diagnostics International, Switzerland). The slides were baked, deparaffinized, pre-treated (CC2, HRP, Inhibitor CM), stained with either primary Rb-Cftr antibody (0.1 µg/mL, #ACL-006, Alemone), Rb-Muc5b antibody (2.5 µg/mL, #PA5-82342, ThermoFisher Scientific) and Rb-GLP-1 receptor antibody (2.7 µg/mL, #218532, Abcam) and enhanced using the Rb-HQ system and HQ-HRP kits (#760-4818 and #760-4820, Roche). The antibodies were labelled using DAB chromogen for single stain protocols, for the dual staining of Muc5b and GLP-1 receptor teal HRP (#769-247, Roche) and yellow AP (#760-239, Roche) combined with the Rb-NP system (#760-4817, #760-4827, Roche) was used, respectively. An identical protocol without a primary antibody was included as a negative control. Protocols were validated by comparing and confirmation of identical locations of in situ hybridization signal and immunohistochemistry signal in adjacent sections. The histologically stained sections were visualized using Nanozoomer s60 whole slide imaging at 40x magnification (Hamamatsu, Japan).

The level of mucin 5b and Cftr-channel was assessed by dividing the DAB-positive area with the number of nuclei in Brunner’s glands. The number of nuclei was determined by segmenting hematoxylin staining using a Visiopharm “AI Nuclei (BF)” algorithm further trained on 8179 nuclei annotations. The DAB-positive pixel area was quantified using HDAB-DAB filtering values below 150 at 20x magnification (8-bit picture format, Visopharm, Hørsholm, Denmark).

#### In situ hybridization

The Ventana Ultra Discovery system was also used for fully automated in situ hybridizations of the Mm-GLP-1 receptor probe (#418859, Advanced Cell Diagnostics), Mm-Cftr probe (#483019, Advanced Cell Diagnostics), Mm-Muc5b probe (#471999, Advanced Cell Diagnostics), Mm-Ren1 probe ( #433469, Advanced Cell Diagnostics) and Mm-Il33 probe (#400599, Advanced Cell Diagnostics). The slides were baked, deparaffinized, pre-treated (target retrieval, inhibitor, protease #323250, Advanced Cell Diagnostics), and in situ hybridization was performed using the RNAscope™ VS Universal AP kit with the VS 2,5 RED reagent kit (#760-236, #760-248, #760-234 Roche, Switzerland). In parallel, a slide was hybridized with DapB (#312039, Advanced Cell Diagnostics) and Ppib (#313919, Advanced Cell Diagnostics), which served as a negative and positive control, respectively. All slides were counterstained with hematoxylin and bluing reagent (Roche), and coverslips were mounted using Pertex mounting medium. The histologically stained sections were visualized using Nanozoomer s60 at 40x magnification (Hamamatsu, Japan). Using the HALO software, a customized algorithm was used to quantify in situ hybridization signal (Indica Labs, Albuquerque, USA). Brunner’s glands were selected as the area of interest for the analysis (n=6-8/grp). Results are shown as H‐score calculated, as recommended by manufacturer, from the equation: H = [1 * (cell fraction with 1–3 dots/cell) + 2 * (cell fraction with 4–9 dots/cell) + 3 * (cell fraction with 10–15 dots/cell) + 4 * (cell fraction with >15 dots/cell)] ^11^.

The mRNA expression (in situ hybridization) was scored using a standard ACD scoring system calculated, as recommended by manufacturer, from the equation: ACD score (1-4) = [1 * (cell fraction with 1–3 dots/cell) + 2 * (cell fraction with 4–9 dots/cell) + 3 * (cell fraction with 10–15 dots/cell) + 4 * (cell fraction with >15 dots/cell)]^11^

[Wang, F., et al., RNAscope: a novel in situ RNA analysis platform for formalin-fixed, paraffin-embedded tissues. J Mol Diagn, 2012. **14**(1): p. 22-9.].

#### In situ hybridization multiplexed with immunofluorescence

Slides were processed on the automated Ventana platform (Roche, Switzerland) as described above using Mm-Malat1 (#313399, Advanced Cell Diagnostics), DapB (#312039, Advanced Cell Diagnostics), and Mm-GLP1r (# 418859, Advanced Cell Diagnostics) probes on adjacent sections containing Brunner’s glands. Immunofluorescence was done by manual staining following the development of the in situ hybridization signal by the VS 2,5 RED reagent kit (#760-236 Roche, Switzerland): Following blocking reagents (#SAT701B001EA, PerkinElmer) primary antibodies, rabbit-anti-smooth muscle actin (#ab5694, Abcam, 1 µg/ml) and mouse-anti-E-cadherin (#610182, BD Transduction Laboratories, 5 µg/ml), were applied for 45 minutes then washed and detected using Brightvision goat-anti-Rb-HRP (#VWRKDPVM110HRP, Immunologic), fluorescein TSA (#SAT701B001EA, PerkinElmer) and donkey anti-mouse-Cy5 (#715-175-151, Jackson ImmunoResearh). The slides were counterstained with 300nM DAPI, then washed and mounted (#S3023, DAKO). The slides were scanned on an Olympus VS120 at 20x magnification (NA 0.75, 0.33μm/pixel) using identical exposure settings for all slides and groups.

*Extended data figures and tables*

**Fig.1**

***Extended Fig.1: Cftr and Mucin 5b immunohistochemical staining in Brunner’s glands.***

*Representative images of Immunohistochemistry of Cftr and Mucin 5b in Brunner’s glands following 4 exposure to liraglutide or vehicle (n=8/grp). Scalebar 250 µm (overview) and 50 µm (zoom).*

**Fig.2**


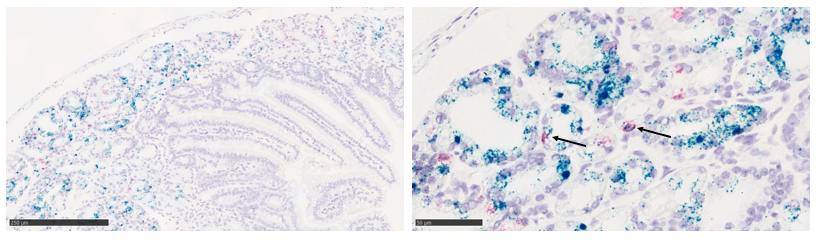


***Extended Fig.2: Ren1 and Muc5b duplex in situ hybridisation in Brunner’s glands.***

*Representative image of duplex in situ hybridisation of Muc5b (blue) and Ren1 (red) after 4 hours of liraglutide. Ren1 was observed in a limited subset of Brunner’s gland cells, while Muc5b was present in a larger number of cells (n=2). Co-expression was observed in very few cells (black arrow). Scalebar 250 µm (left) and 50 µm (right).*

**Fig.3**

**Extended Fig.3: *Cftr and Mucin 5b immunohistochemical staining in Brunner’s glands in GLP-1R KO mice.***

*Representative images of Cftr and Mucin 5b stained by immunohistochemistry in Brunner’s glands of GLP-1R KO mice (n=2).*

1. Scrocchi, L.A.*, et al*. Glucose intolerance but normal satiety in mice with a null mutation in the glucagon-like peptide 1 receptor gene. *Nat Med*. **2**, 1254-1258 (1996).

2. Ämmälä, C.*, et al*. Targeted delivery of antisense oligonucleotides to pancreatic β-cells. *Science advances*. **4**, eaat3386 (2018).

3. Andrews, S. FastQC: A Quality Control Tool for High Throughput Sequence Data. 2010.

4. Hannon, G.J. FASTX-Toolkit. 2010.

5. Dobin, A.*, et al*. STAR: ultrafast universal RNA-seq aligner. *Bioinformatics*. **29**, 15-21 (2012).

6. Team, R.C. R: A language and environment for statistical computing. . 2018.

7. Kinsella, R.J.*, et al*. Ensembl BioMarts: a hub for data retrieval across taxonomic space. *Database : the journal of biological databases and curation*. **2011**, bar030 (2011).

8. Love, M.I., Huber, W. & Anders, S. Moderated estimation of fold change and dispersion for RNA-seq data with DESeq2. *Genome biology*. **15**, 550 (2014).

9. Yu, G., Wang, L.G., Han, Y. & He, Q.Y. clusterProfiler: an R package for comparing biological themes among gene clusters. *Omics : a journal of integrative biology*. **16**, 284-287 (2012).

10. Carlson, M. org.Hs.eg.db: Genome wide annotation for human. 2019. R package version 3.10.0. 2019.

11. Wang, F.*, et al*. RNAscope: a novel in situ RNA analysis platform for formalin-fixed, paraffin-embedded tissues. *J Mol Diagn*. **14**, 22-29 (2012).
